# Azoramide protects iPSC-derived dopaminergic neurons with PLA2G6 D331Y mutation through restoring ER function and CREB signaling

**DOI:** 10.1038/s41419-020-2312-8

**Published:** 2020-02-18

**Authors:** Minjing Ke, Cheong-Meng Chong, Haitao Zeng, Miaodan Huang, Zhijian Huang, Ke Zhang, Xiaotong Cen, Jia-Hong Lu, Xiaoli Yao, Dajiang Qin, Huanxing Su

**Affiliations:** 1 State Key Laboratory of Quality Research in Chinese Medicine, Institute of Chinese Medical Sciences, University of Macau, Macao, China; 2 Center for Reproductive Medicine, the Sixth Affiliated Hospital of Sun Yat-Sen University, Guangzhou, 510080 China; 3 Guangzhou Regenerative Medicine and Health Guangdong Laboratory; The Fifth Affiliated Hospital of Guangzhou Medical University, Guangzhou, China; 4 South China Institute for Stem Cell Biology and Regenerative Medicine, Guangzhou Institute of Biomedicine and Health, Chinese Academy of Sciences, Guangzhou, China; 5 Department of Neurology, National Key Clinical Department and Key Discipline of Neurology, the First Affiliated Hospital of Sun Yat-Sen University, Guangzhou, 510080 China

**Keywords:** Molecular neuroscience, Parkinson's disease

## Abstract

The endoplasmic reticulum (ER)-stress-induced cascade events are implicated in Parkinson’s disease (PD). The discovery of drug candidates to protect dopaminergic (DA) neurons from ER-stress-induced oxidative damage is important to resolve the pathological aspects of PD and modify its progress. In this study, we found that a recently identified unfolded protein response (UPR) modulator, azoramide, showed protective effects on patient induced pluripotent stem cells-derived midbrain DA neurons with the homozygous phospholipase A2 group 6 (PLA2G6) D331Y mutant. A series of PD-related cascade events such as ER stress, abnormal calcium homeostasis, mitochondrial dysfunction, increase of reactive oxygen species, and apoptosis were observed in PLA2G6 D331Y mutant DA neurons, whereas azoramide significantly protected PLA2G6 D331Y mutant DA neurons against these events. The beneficial effects of azoramide were abolished by treatment with a cAMP-response element binding protein (CREB) inhibitor. Our results suggest that azoramide is a potential neuroprotectant against DA neuron damage via restoring ER function and the CREB signaling.

## Introduction

Parkinson’s disease (PD) is the second most common age-related neurodegenerative disorder. It is mainly caused by the progressive loss of dopaminergic (DA) neurons in the substantia nigra pars compacta (SNpc) of the midbrain. The loss of DA neurons leads to a decrease in dopamine, which is a key neurotransmitter for the coordination of motor control and behavior. Accordingly, PD is clinically characterized by motor symptoms called parkinsonism such as tremor, rigidity, bradykinesia, loss of facial expression, and balance problems^1–3^. At present, the etiology and pathogenesis of PD are poorly understood. The treatment options like medication and surgery only relieve PD patients’ symptom, and there is no effective therapy to slow or halt the progressive loss of midbrain DA neurons in PD.

Although the etiology of PD remains unknown, increasing evidence supports the critical role of endoplasmic reticulum (ER) stress in the pathogenesis of PD^4,5^. The ER is the main subcellular organelle involved in protein folding, maturation, and quality control. The accumulation of unfolded or misfolded proteins in the ER is known to induce ER stress, resulting in the unfolded protein response (UPR), which serves as an ER stress sensor or transducer. UPR initially restores normal ER function via reducing protein translation, degrading misfolded proteins, and activating signaling pathways to increase expression of molecular chaperones for protein folding^6,7^. However, if ER stress cannot be restored by UPR and is prolonged, apoptosis is triggered. Notably, normal ER function is important to maintain Ca^2+^ homeostasis, redox balance, and mitochondrial function^8–11^. Thus, ER stress is considered to be a potential upstream target that plays a critical role in regulating the survival of DA neurons in PD.

Familial PD (FPD) is caused by mutations in specific genes (*PARKs*), which accounts for 10% of PD cases^12^. Several *PARKs* mutations have been linked to ER stress, mitochondrial dysfunction, α-synuclein (α-syn) accumulation, and other cellular defects, which are characteristics of PD^13^. A homozygous c.991 G>T (Asp331Tyr, D331Y) mutation in phospholipase A2 group 6 (PLA2G6) gene at the *PARK14* locus is known to cause the common PD pathology and triggers PD-related motor symptoms^14–16^. Increasing evidence suggests that the PLA2G6 D331Y mutant triggers a distinct loss of DA neurons, accompanied by accumulated ER stress, mitophagy dysfunction, and reactive oxygen species (ROS) generation^17^. In the present study, we established patient-derived induced pluripotent stem cells (iPSCs) with homozygous PLA2G6 D331Y mutation, and further differentiated them into midbrain DA neurons. Using the PLA2G6 D331Y mutant DA neuron-based PD model, we identified azoramide, a modulator of UPR, as a protector against apoptosis of degenerating midbrain DA neurons. We found that azoramide protected midbrain DA neurons against apoptosis through reducing abnormal ER-mediated Ca^2+^ homeostasis, ROS increase, mitochondrial membrane potential decline, and caspase 3 activation, suggesting that azoramide is a potential neuroprotectant against ER-stress-induced PD cascade events.

## Results

### Characterization of FPD PLA2G6 D331Y mutant iPSC-derived midbrain DA neurons

FPD PLA2G6^D331Y/D331Y^ patient-derived iPSCs were established by reprogramming the urine cells from a male patient donor as described previously^18^. Immunostaining showed that PLA2G6^D331Y/D331Y^ iPSCs displayed the pluripotent markers Oct4, Nanog, and Sox2 and exhibited normal karyotypes and the ability to form teratomas containing the tissues of all three germ cell layers (Supplementary Fig. 1). Sanger sequencing demonstrated that these PLA2G6^D331Y/D331Y^ iPSCs carried the homozygous autosomal recessive missense mutation (D331Y) in exon 7 of PLA2G6 (Supplementary Fig. 1).

**Fig. 1:**
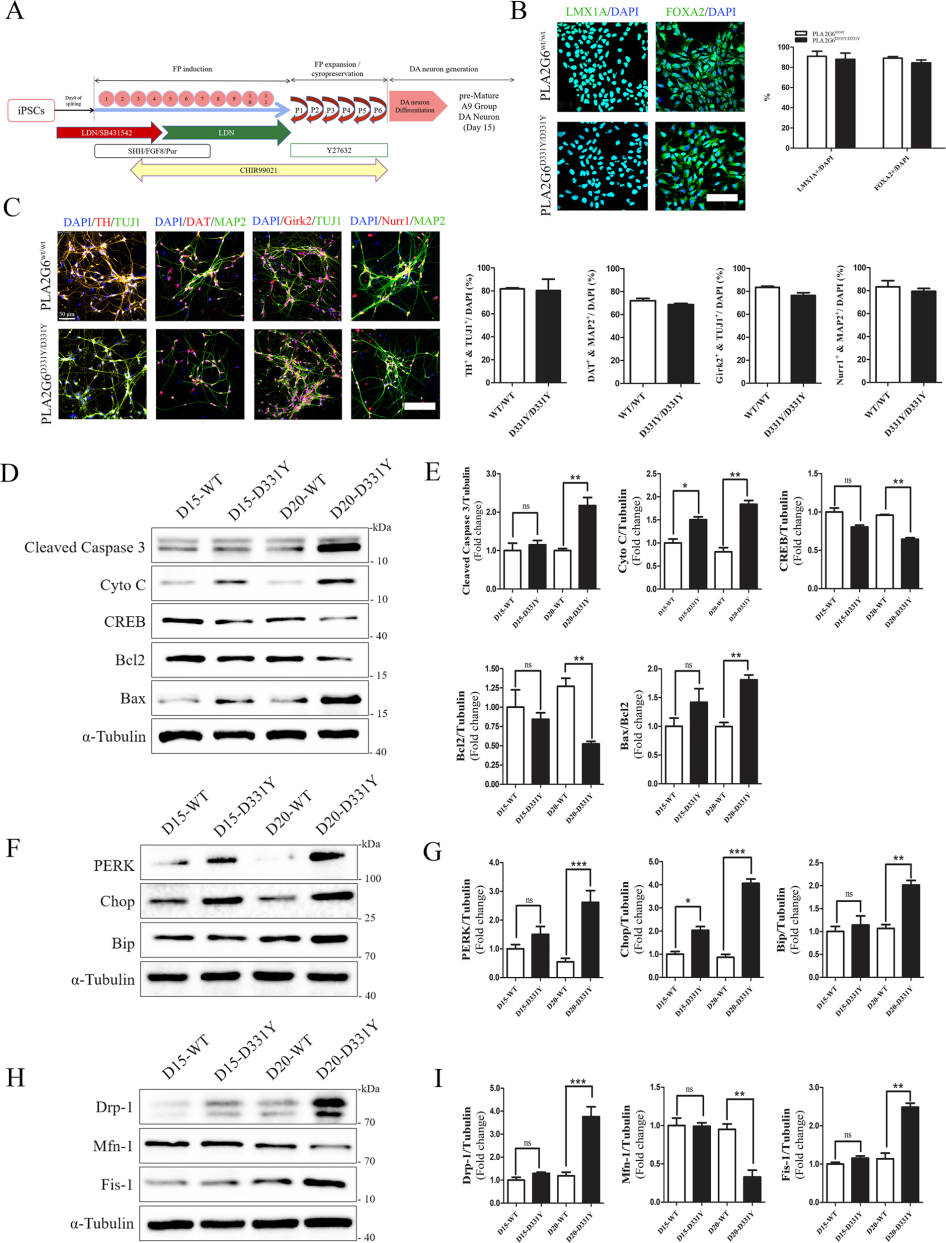
Characterization of FPD PLA2G6 D331Y mutant iPSC-derived midbrain DA neurons and time course of cell fate. **a** Schematic diagram of generation of DA neurons. iPSCs were gradually exposed to different small molecules, inducing midbrain characteristics, and finally determining dopaminergic fate of DA neurons. iPSCs were first transformed into FP cells and after 6 passages the culture medium was changed to induce DA neurons. **b** LMX1A and FOXA2 immunostaining of FP cells. **c** TH, Girk2, Nurr1 and DAT immunostaining of day 15 DA neurons exhibited midbrain features. **d**, **e** Time course of apoptosis of DA neurons. Expression of apoptosis-related proteins, including cleaved caspase 3, cytochrome C, Bax, Bcl2 and CREB, was detected and quantified by western blotting using specific antibodies. **f**, **g** Time course of ER stress on DA neurons. Expression of ER stress protein was determined and quantified by Western blotting. **h**, **i** Time course of mitochondrial function in DA neurons. α-Tubulin was the internal control. Data are represented as mean ± SEM. All experiments were replicated in triplicate independently. *p < 0.05, **p < 0.01, ***p < 0.005. Scale bar: 30 µm for **b** and 50 µm for **c**.

Using a well-established midbrain DA neuron differentiation protocol with some minor modifications^19,20^, we successfully generated the floor plate (FP) cells and mature A9 group DA neurons from control and PLA2G6^D331Y/D331Y^ iPSCs (Fig. 1a). Human UC-H1-iPSCs established in our previous study served as the controls^19,20^. Group A9 is the most densely packed group of DA neurons in the ventrolateral midbrain. Immunostaining showed that FP cells differentiated from both the control and PLA2G6^D331Y/D331Y^ iPSCs were positive for LMX1A and FOXA2 (Fig. 1b). These FP cells were further differentiated into DA neurons expressing the dopaminergic biomarkers TH, DAT, Girk2, and Nurr1 (Fig. 1c). The yield of FP cells and DA neurons was similar between the control and PLA2G6^D331Y/D331Y^ iPSCs (Fig. 1c), suggesting that PLA2G6 D331Y mutant did not affect the differentiation of DA neurons. Western blotting revealed that cytochrome C was released from mitochondria and the apoptosis pathway was subsequently activated in D331Y mutant DA neurons after culture for 15 and 20 days (Fig. 1d, e). cAMP response element binding protein (CREB) expression was significantly decreased in D331Y mutant DA neurons (Fig. 1d, e). The ER-stress-related proteins were markedly accumulated in D331Y mutant DA neurons compared with healthy control neurons after culture for 15 and 20 days (Fig. 1f, g), which was consistent with the previous report that mutant PLA2G6 could lead to elevated UPR^17^. Abnormal expression of mitochondrial proteins (upregulation of fission proteins Drp-1 and Fis-1 and downregulation of fusion protein Mfn-1) was detected in PLA2G6 mutant neurons after culture for 20 days (Fig. 1h, i). We also generated non-dopaminergic neurons from PLA2G6^D331Y/D331Y^ iPSCs which were TH-negative but MAP2-positive. No obvious alterations in ER stress-related proteins, intracellular ROS level, and mitochondrial proteins were found in non-dopaminergic neurons (Supplemental Fig. 2), suggesting that PLA2G6 mutant may selectively cause ER stress and mitochondrial dysfunction in dopaminergic neurons.

### Azoramide attenuates loss of PLA2G6^D331Y/D331Y^ iPSC-derived midbrain DA neurons

**Fig. 2:**
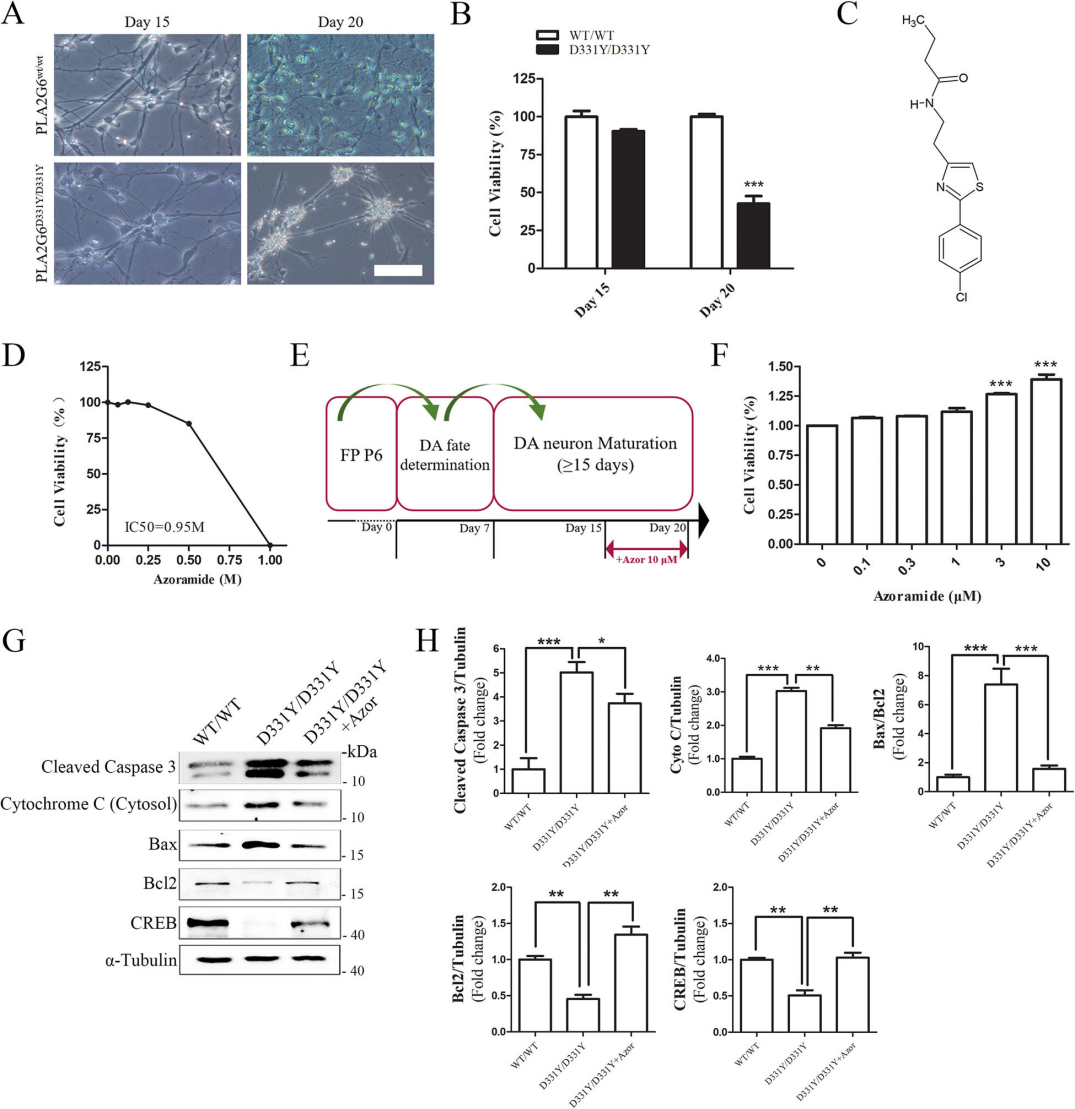
Azoramide alleviates apoptosis of PLA2G6^D331Y/D331Y^ DA neurons. **a** Representative bright field view of PLA2G6^WT/WT^ and PLA2G6^D331Y/D331Y^ DA neurons at days 15 and 20. **b** Cell viability of PLA2G6^WT/WT^ and PLA2G6^D331Y/D331Y^ after 15 and 20 days differentiation culture was measured by CCK-8 assay. **c** Structure of azoramide. **d** PLA2G6^D331Y/D331Y^ neurons at day 15 were treated with azoramide (0.0625, 0.125, 0.25, 0.5, 1 M) or 0.1% DMSO (vehicle control) for 24 h and cell viability was measured using the CCK-8 assay. **e** Schematic diagram of drug treatment process. **f** PLA2G6^D331Y/D331Y^ DA neurons were treated with azoramide (0.1, 0.3, 1, 3, 10 μM) from day 15 to 20, and viability was determined by CCK-8 assay. **g**, **h** Expression of apoptosis-related proteins, including cleaved caspase 3, cytochrome C, Bax, Bcl2 and CREB, was detected and quantified by western blotting using specific antibodies. α-Tubulin was the internal control. Data are represented as mean ± SEM. All experiments were replicated in triplicate independently. *p < 0.05, **p < 0.01, ***p < 0.005. Scale bar: 50 µm.

After differentiation into mature DA neurons, we compared the viability of PLA2G6^D331Y/D331Y^ DA and control DA neurons using the CCK-8 assay. No significant decrease in cell viability was found between the control and PLA2G6 D331Y group after culture for 15 days (Fig. 2a, b); however, the viability of PLA2G6^D331Y/D331Y^ DA neurons was significantly decreased (57%) after culture for 20 days compared with the controls, revealing that PLA2G6 D331Y mutant caused neuronal death. To evaluate the cytotoxicity of azoramide (Fig. 2c), PLA2G6^D331Y/D331Y^ midbrain DA neurons were incubated with various concentrations of azoramide for 24 h. Azoramide up to 0.25 M did not cause any cytotoxicity in PLA2G6^D331Y/D331Y^ DA neurons (Fig. 2d). The IC50 of azoramide (0.95 M) was higher than 10 μM, indicating the safety of azoramide. DA neurons were treated with various concentrations of azoramide on day 15 of culture and the treatment lasted for 5 days (Fig. 2e). CCK-8 assay showed that 3 and 10 μM azoramide significantly enhanced cell viability (27 and 39%, respectively) (Fig. 2e). Therefore, 10 μM was used as a working concentration of azoramide for neuroprotection. Western blotting demonstrated that 10 μM azoramide dramatically inhibited the release of cytochrome c from mitochondria and decreased the cleaved level of caspase 3 and the ratio of Bax/Bcl2 in PLA2G6 mutant neurons (Fig. 2g, h). Azoramide significantly enhanced expression of CREB in PLA2G6 mutant neurons (Fig. 2g, h).

### Azoramide reduces the increase in ROS and ameliorates the decline in mitochondrial membrane potential in PLA2G6^D331Y/D331Y^ midbrain DA neurons

**Fig. 3:**
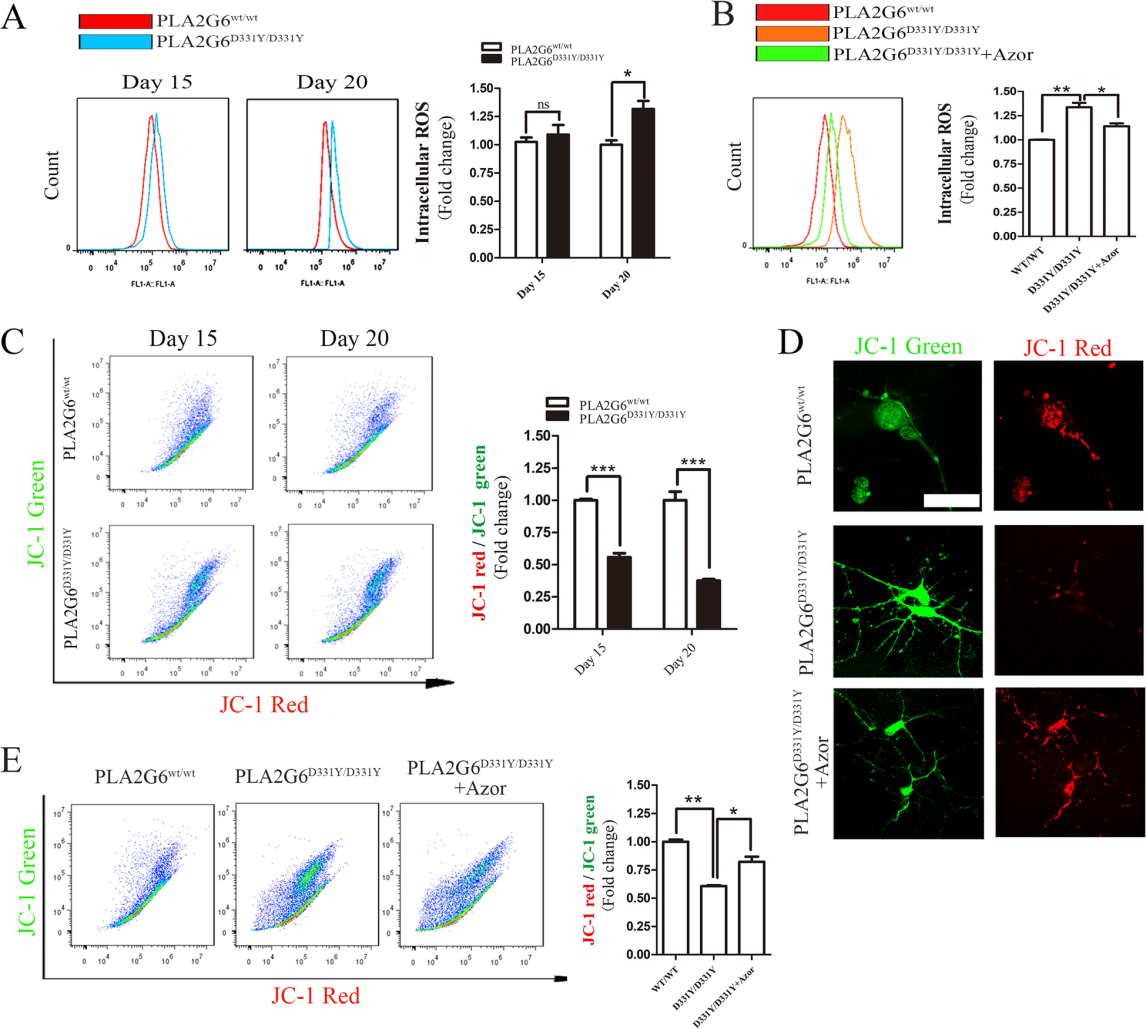
Azoramide reduces mitochondrial dysfunction in PLA2G6^D331Y/D331Y^ DA neurons. **a** Intracellular ROS levels of PLA2G6^WT/WT^ and PLA2G6^D331Y/D331Y^ DA neurons at day 15 and 20 were measured by the fluorescent probe CellROX® Green Reagent and flow cytometry. **b** After PLA2G6^D331Y/D331Y^ DA neurons were treated with 10 μM azoramide from day 15 to 20, intracellular ROS levels were assessed by CellROX® Green Reagent and flow cytometry. **c** Mitochondrial membrane potential of PLA2G6^WT/WT^ and PLA2G6^D331Y/D331Y^ DA neurons at day 15 and 20 was determined by JC-1 staining and flow cytometry. **d** After PLA2G6^D331Y/D331Y^ DA neurons were treated with 10 μM azoramide from day 15 to 20, mitochondrial membrane was stained by JC-1. **e** Mitochondrial membrane potential was determined by flow cytometry. Data are represented as mean ± SEM. All experiments were replicated in triplicate independently. *p < 0.05, **p < 0.01, ***p < 0.005. Scale bar: 30 µm.

Oxidative stress causes the release of cytochrome c from mitochondria, which activates the apoptotic pathway^21–25^. Intracellular ROS in DA neurons was measured with the fluorescent probe CellROX® Green Reagent. The intracellular level of ROS was not different between the PLA2G6 mutant DA neurons and control neurons after culture for 15 days (Fig. 3a). Notably, it was significantly elevated after culture for 20 days compared with control neurons (Fig. 3a). The increase in intracellular ROS was reduced by treatment with 10 μM azoramide (Fig. 3b). Oxidative stress and mitochondrial dysfunction interact closely^21^. The loss of mitochondrial membrane potential is a typical hallmark of mitochondrial dysfunction. We then assessed the mitochondrial membrane potential in DA neurons by analyzing the red/green fluorescent intensity ratio of JC-1 staining. Compared with control neurons, PLA2G6 mutant DA neurons displayed decreased JC-1 red/green ratios after culture for 15 and 20 days (Fig. 3c), revealing that mitochondrial dysfunction occurred prior to ROS increase in PLA2G6 mutant DA neurons. Treatment with 10 μM azoramide enhanced JC-1 red intensity (Fig. 3d), thereby rescuing the decline in JC-1 red/green ratio in PLA2G6 mutant DA neurons (Fig. 3e). This suggests that azoramide attenuates the loss of PLA2G6 mutant-induced mitochondrial membrane potential.

### Azoramide suppresses mitochondrial fragmentation in PLA2G6^D331Y/D331Y^ midbrain DA neurons

**Fig. 4:**
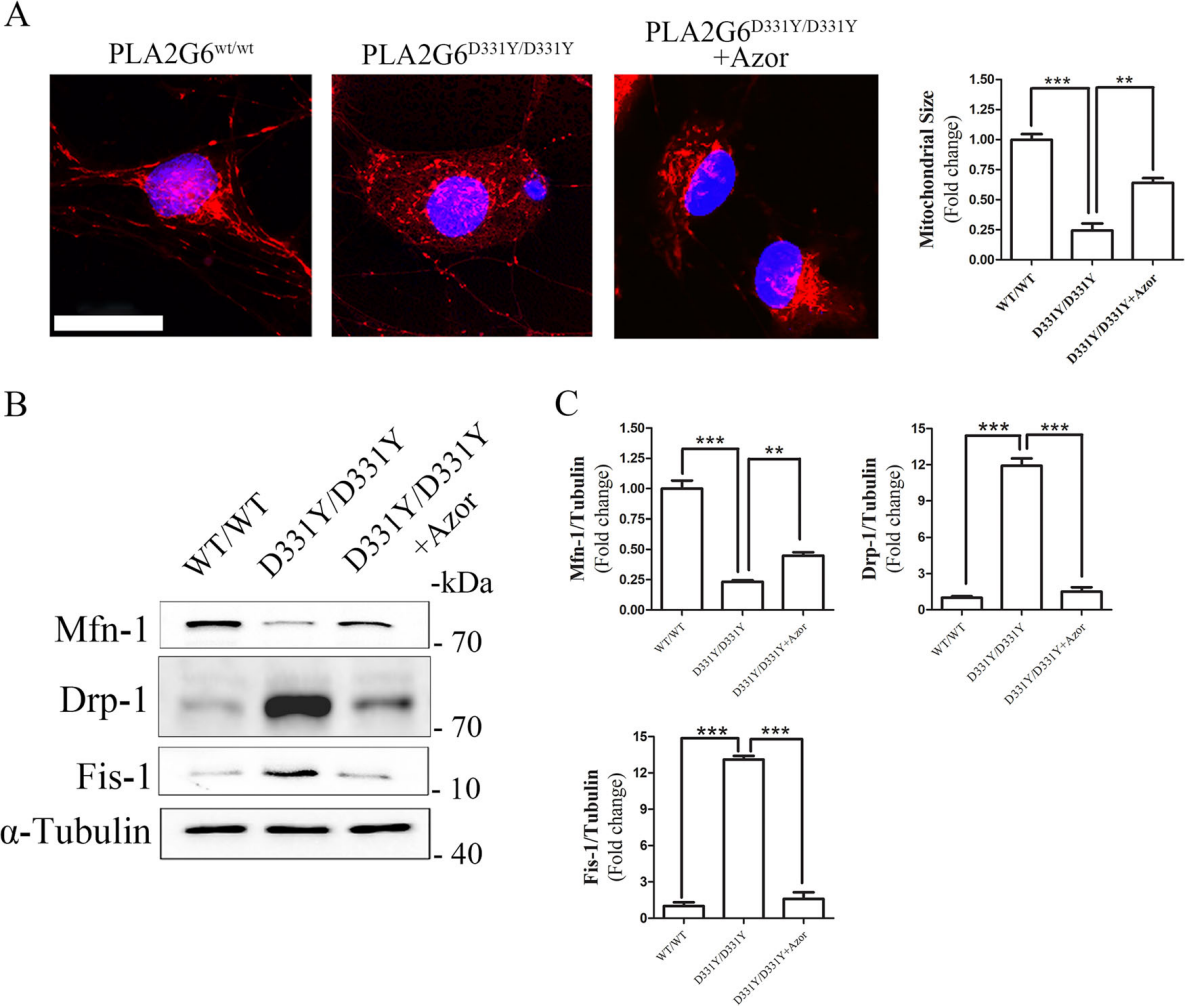
Azoramide prevents mitochondrial fragmentation in PLA2G6^D331Y/D331Y^ DA neurons. **a** PLA2G6^D331Y/D331Y^ DA neurons were treated with 10 μM azoramide from day 15 to 20. Representative images and quantification of mitochondria in PLA2G6^WT/WT^, PLA2G6^D331Y/D331Y^ and azoramide-treated PLA2G6^D331Y/D331Y^ DA neurons after MitoTracker Red staining. **b**, **c** Expression of mitochondrial fission and fusion proteins was determined and quantified by western blotting. α-Tubulin was the internal control. Data are represented as mean ± SEM. **p < 0.01, ***p < 0.005. Scale bar: 20 µm.

Mitochondrial fragmentation has been observed to accompany oxidative damage in neurons^26^. Using MitoTracker Red staining, we found dominant mitochondrial fragmentation in PLA2G6 mutant DA neurons after culture for 20 days (Fig. 4a). In azoramide-treated PLA2G6 mutant DA neurons, mitochondrial fragmentation was dramatically reduced (Fig. 4a). Western blotting showed that azoramide enhanced the decreased expression level of mfn1 and suppressed the elevated expression levels of DRP1 and Fis1 found in PLA2G6 mutant DA neurons, compared with control neurons (Fig. 4b, c). We also valuated the effects of Azoramide on protecting PLA2G6 mutant dopaminergic neurons after culture for 30 days. Western blotting demonstrated that Azoramide significantly decreased the cleaved level of caspase 3 and the ratio of Bax/Bcl2 and enhanced the expression of CREB. Meanwhile, Azoramide significantly suppressed increased expression of UPR proteins, elevated the decreased expression level of mfn1, and inhibited the elevated expression levels of DRP1 and Fis1 in PLA2G6 mutant neurons after culture for 30 days (Supplementary Fig. 3).

### Azoramide restores Ca^2+^ homeostasis and reduces UPR in PLA2G6^D331Y/D331Y^ midbrain DA neurons

**Fig. 5:**
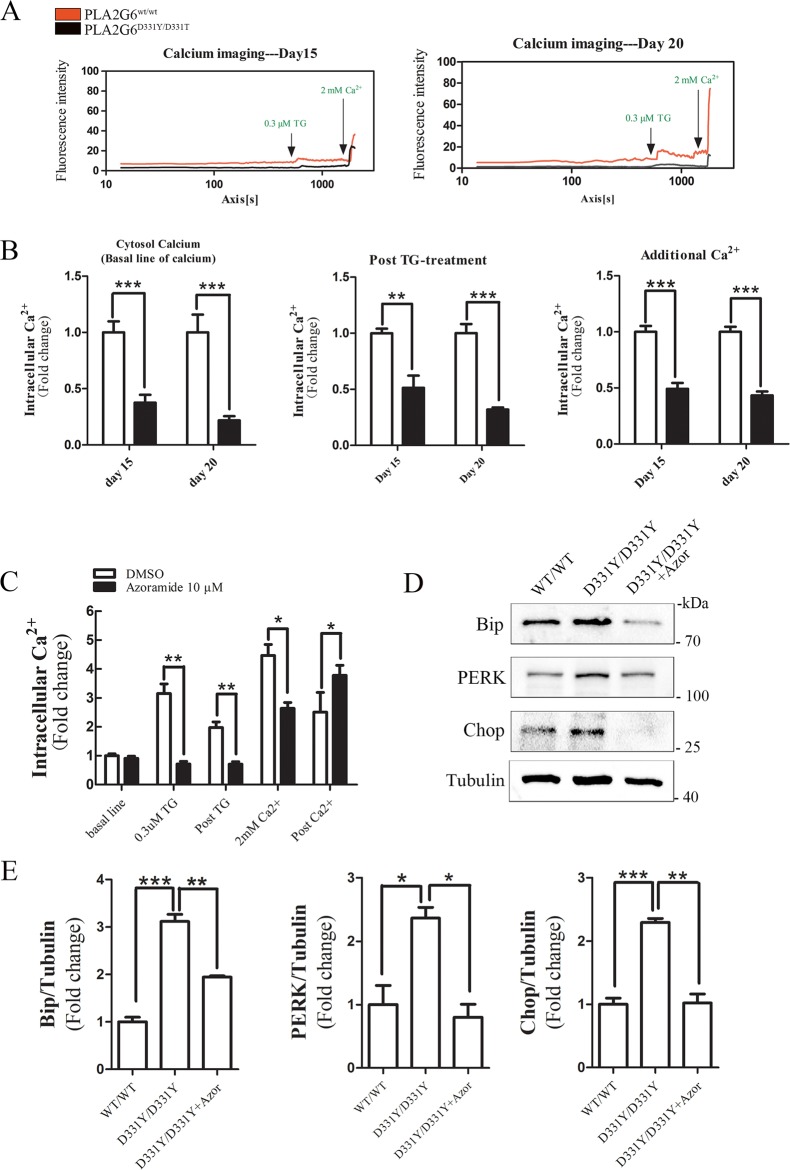
Azoramide restores Ca^2+^ homeostasis and decreases ER stress in PLA2G6^D331Y/D331Y^ DA neurons. **a** Time-lapse fluorescence changes of intracellular Ca^2+^ levels of PLA2G6^WT/WT^ and PLA2G6^D331Y/D331Y^ DA neurons at day 15 and 20 were determined by Fluo-4 AM. Representative results of calcium imaging. **b** Intracellular Ca^2+^ levels at baseline, post-TG treatment, and after additional Ca^2+^ treatment in PLA2G6^WT/WT^ and PLA2G6^D331Y/D331Y^ DA neurons. **c** PLA2G6^D331Y/D331Y^ DA neurons were treated with 10 μM azoramide from day 15 to 20. Changes in intracellular Ca^2+^ levels were measured by calcium imaging. **d**, **e** Expression of ER stress protein was determined and quantified by western blotting. α-Tubulin was the internal control. Data are represented as mean ± SEM. All experiments were replicated in triplicate independently. **p < 0.01, ***p < 0.005. Scale bar: 30 µm.

Store operated Ca^2+^ entry (SOCE) is important for Ca^2+^ homeostasis in the cytosol and ER (Supplementary Fig. 2). PLA2G6 is reported to function in regulating SOCE^27–30^. To assess the effects of PLA2G6 mutant on SOCE, a time course measurement was performed to detect the dynamic changes of intracellular Ca^2+^ using a Ca^2+^-binding probe Fluo-4 AM. The sarco/ER Ca^2+^ ATPase (SERCA) inhibitor thapsigargin (TG), induced an acute Ca^2+^ depletion from ER, thereby activated SOCE and induced Ca^2+^ influx in control neurons and PLA2G6 mutant neurons (Fig. 5a). However, the levels of intracellular Ca^2+^ at baseline and post-TG treatment in control neurons were higher than in PLA2G6 mutant neurons (Fig. 5a, b). SOCE was activated by Ca^2+^ leakage in the ER^31^. Exogenous Ca^2+^ was subsequently added to evaluate SOCE functions. The increased level of Ca^2+^ in control neurons was higher than in PLA2G6 mutant DA neurons after adding exogenous Ca^2+^ (Fig. 5a, b). These results suggest that PLA2G6 mutant caused the dysfunction of SOCE and ER. In contrast, azoramide treatment enhanced SOCE function to increase Ca^2+^ level in PLA2G6 mutant DA neurons (Fig. 5c), suggesting that azoramide restored Ca^2+^ homeostasis in PLA2G6 mutant DA neurons via mediating SOCE and ER function. Western blotting showed that azoramide significantly suppressed increased expression of UPR proteins in PLA2G6 mutant DA neurons (Fig. 5d, e), suggesting that azoramide restores ER function.

### CREB is involved in the protective effect of azoramide in PLA2G6^D331Y/D331Y^ midbrain DA neurons

**Fig. 6:**
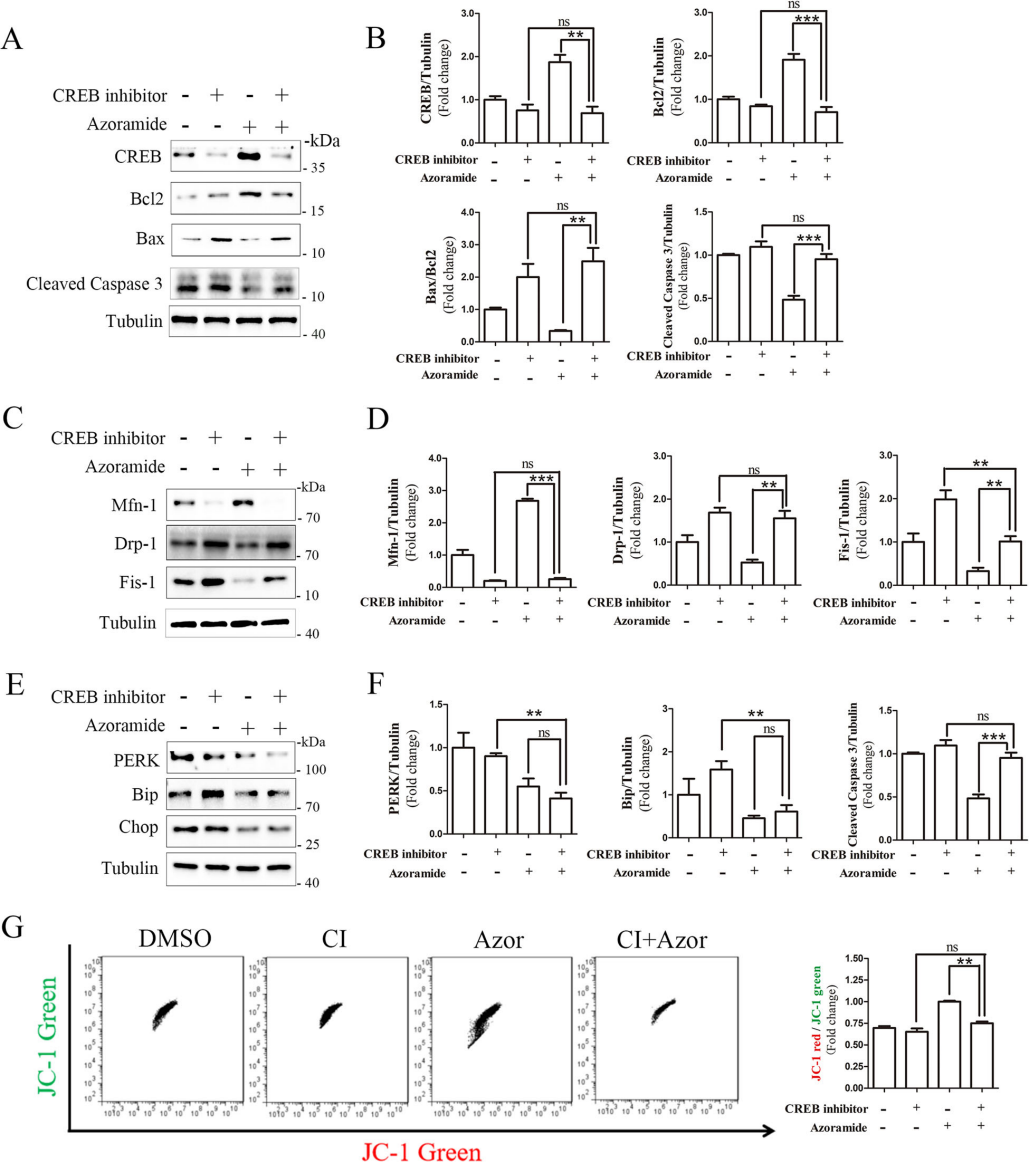
CREB inhibitor blocks the effects of azoramide in PLA2G6^D331Y/D331Y^ neurons. **a**, **b** PLA2G6^D331Y/D331Y^ DA neurons were treated with 10 μM azoramide or 1 μM CREB inhibitor 666-15, or both, from day 15 to 20. Expression of apoptosis-related proteins was determined and quantified by western blotting. **c**, **d** Expression of mitochondrial fission- and fusion-related proteins. **e**, **f** Expression of ER-stress-related proteins. **g** Mitochondrial membrane potential was determined by flow cytometry after JC-1 staining. Data are represented as mean ± SEM. All experiments were replicated in triplicate independently. *p < 0.05, **p < 0.01, ***p < 0.005.

Bcl2 is a stabilizer of the mitochondrial membrane, which is regulated by a key transcript factor CREB^32^. Azoramide restored CREB and Bcl2 expression (Fig. 2g); however, it is unknown whether the CREB/Bcl2 pathway is involved in the neuroprotective effects of azoramide. CREB inhibitor 666-15 significantly reduced the enhancing effects of azoramide on expression of CREB and Bcl2 in PLA2G6 mutant neurons, and attenuated the suppressive effects of azoramide on expression of cleaved caspase 3 and Bax (Fig. 6a, b). This suggests that CREB signaling is involved in the protective effects of azoramide against apoptosis. The CREB inhibitor also suppressed the effects of azoramide on expression of Mfn-1, Drp-1 and Fis-1, suggesting that the CREB signaling is involved in the effects of azoramide on mitochondrial fragmentation (Fig. 6c, d). However, the CREB inhibitor did not attenuate the effects of azoramide on expression of PERK, Bip and Chop, suggesting that ER stress may be upstream of CREB signaling (Fig. 6e, f). The CREB inhibitor did not alter the enhancing effects of azoramide on Ca^2+^ level in PLA2G6 mutant DA neurons (data now shown). JC-1 staining revealed that the CREB inhibitor blocked the effects of azoramide on the ratio of JC-1 red/green, confirming that CREB signaling is involved in the effects of azoramide on improving mitochondrial function (Fig. 6g). These results suggest that azoramide protects DA neurons against apoptosis through restoring the ER function and the CREB signaling.

## Discussion

Sustained ER stress disrupts normal UPR and mitochondrial functions, thereby causing irreversible oxidative damage to DA neurons^33^. In this study, we established a model based on PLA2G6 mutant, patient-iPSC-derived, midbrain DA neurons, and found that the PLA2G6 mutant caused PD-related cascade events in DA neurons, such as imbalance of Ca^2+^ homeostasis, increase of UPR proteins, decline of mitochondrial membrane potential, fragmentation of mitochondria, increase of ROS, and apoptosis, which was consistent with previous reports^27,34–38^. We for the first time reported that an UPR modulator azoramide was able to significantly suppress these abnormal changes in PLA2G6 mutant DA neurons.

Azoramide was originally identified to modulate ER folding activity and UPR, and displays potent antidiabetic activity in vivo through improving insulin sensitivity and pancreatic β cell function^39^. In this study, we found that the basal cytosolic Ca^2+^ level in PLA2G6 mutant DA neurons was lower than in control neurons. Generally, ER function plays an important role in maintaining Ca^2+^ homeostasis. The decline in ER Ca^2+^ results in ER stress, thereby causing UPR. Compared with the control neurons, the level of UPR proteins increased in PLA2G6 mutant DA neurons, suggesting that PLA2G6 D331Y mutant causes ER stress and abnormal UPR. Our results showed that azoramide enhanced SOCE function in PLA2G6 mutant DA neurons. Simultaneously, the increase of UPR proteins was reduced by azoramide, revealing that azoramide reduces ER stress and restores the ER functions in PLA2G6 mutant DA neurons. Therefore, azoramide is a potential drug candidate to modulate ER function, and may be beneficial for treatment of PD.

Mitochondrial dysfunction is one of the major dysfunctional changes in PD patients^40^. Several PD-related neurotoxins such as 6-OHDA, MPP^+^ and rotenone also target mitochondria to block the electron transport chain, and then trigger generation of ROS. It has been reported that the strategies used to stabilize mitochondrial functions have therapeutic potential to prevent DA neuronal death. Our results showed that PLA2G6 D331Y mutant-induced loss of mitochondrial membrane potential, increased ROS, and activation of the mitochondrial apoptosis pathway could be suppressed by azroamide. In addition, changes in mitochondrial morphology are associated with cell health^41,42^. Mitochondrial fragmentation was observed in PLA2G6 D331Y mutant DA neurons, whereas azoramide maintained normal mitochondrial morphology in PLA2G6 D331Y mutant DA neurons. These results provide evidence to support the dominant mitochondrial protective activity of azoramide.

CREB is a central signaling component that plays a role in the initiation and regulation of most cellular processes in neurons, particularly mitochondrial functions^43^. In this study, we observed that levels of CREB and Bcl2 in PLA2G6 mutant DA neurons were significantly lower than in control neurons, revealing that PLA2G6 mutant decreases signaling of CREB. This may result from the abnormal UPR in PLA2G6 mutant DA neurons because abnormal UPR can block most protein translation. In addition, cytosol Ca^2+^ is the upstream signal for positive regulation of the activity of CREB^44^. SOCE is important for maintaining cytosol and ER Ca^2+^ levels^45^; however, PLA2G6 D331Y mutant impaired SOCE and caused lower cytosol Ca^2+^ level. Our results demonstrate that azoramide could rescue SOCE to increase cytosol Ca^2+^, which may contribute to activation of CREB signaling. Furthermore, our results showed that azoramide failed to suppress apoptosis, changes in mitochondrial-fragmentation-related proteins, and loss of mitochondrial membrane potential in the presence of CREB inhibitor 666-15. However, CREB inhibitor did not block the ER-modulating activity of azoramide, revealing that ER stress is an upstream event prior to the decline of CREB activity in PLA2G6 D331Y mutant DA neurons.

**Fig. 7:**
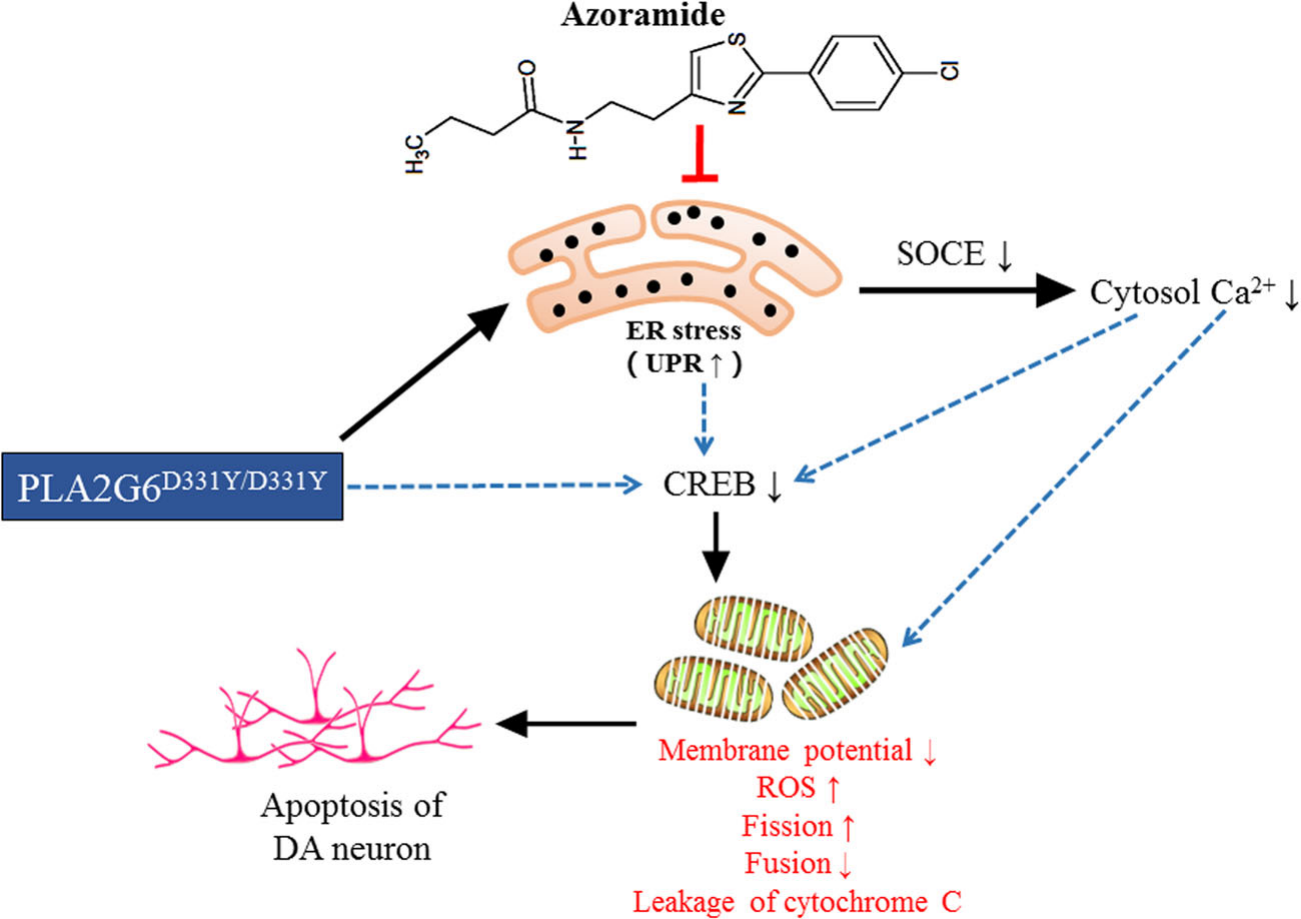
A proposed mechanism of action of azoramide. In PLA2G6 D331Y mutant DA neurons, mutant PLA2G6 impairs ER to trigger UPR, and causes disruption of SOCE-mediated Ca^2+^ influx, resulting in the decreased cytosol Ca^2+^ level. ER stress, cytosol Ca^2+^ imbalance, or PLA2G6 mutant may act together or independently to decrease the expression of CREB, which leads to mitochondrial dysfunction including ROS accumulation, decline of mitochondrial membrane potential, mitochondrial fragmentation, and eventually triggers apoptosis. Azoramide alleviates PLA2G6 mutant-induced ER stress through modulating UPR, and enhances the CERB signaling to rescue mitochondrial function, thereby preventing apoptosis of DA neurons.

In summary, our results provide mechanistic evidence to support the notion that azoramide protects PLA2G6 D331Y mutant DA neurons against degeneration through reducing ER stress, restoring Ca^2+^ homeostasis, enhancing CERB signaling to rescue mitochondrial function (Fig. 7). The promising effects of azoramide suggest that using small molecules to modulate UPR and alleviate ER stress is a worthwhile strategy for the treatment of PD. However, it should be noted that all the beneficial effects of azoramide are based on a single mutant iPSC line. To better address whether azoramide is more broadly valuable to treat PD, more iPS cell lines from PD patient with different mutations are needed to test the effects of azoramide on reducing ER stress and improving mitochondrial defects in the future.

## Materials and methods

### Materials

Azoramide (Cat# SYA965090) was purchased from Toronto Research Chemicals. For immunostaining, DAPI (Cat# C1005) was obtained from Beyotime. LMX1A (Cat# HPA030088) was from Atlas. FOXA (Cat# 22474-1-AP) was from Proteintech. DAT (Cat# MAB369) was from Millipore. TH (Cat# P40101-150) was obtained from Pel Freez. Girk2 (Cat# APC-006) was obtained from Alomone Laboratories. Nurr1 (Cat# PP-N1404-00) was from R&D. MAP2 (Cat# 4542S) was from Cell Signaling Technology. TUJ1 (Cat# T8578) was obtained from Sigma. For Western blotting, cleaved caspase 3 (Cat# 9961S), cytochrome C (Ca# 11940), Bax (Cat# 2772S), Bcl-2 (Cat# 2870S), PERK (Cat# 3192), Drp-1 (Cat# 8570S), Mfn-1 (Cat# 14739S), CHOP (Cat# 2895) and CREB (Cat# 9197S) were purchased from Cell Signaling Technology. Fis-1 (Cat# PA5-22142) was from Invitrogen. Bip (Cat# ab21685) was obtained from Abcam.

### Establishment of FPD patient iPSCs

The generation of human iPSCs from urine cells followed our previously reported protocol^18^. Urine cells were gathered from the donors with FPD PLA2G6^D331Y/D331Y^ mutant (with informed consent) based on Institutional Review Board approval. A total of ~500 mL of urine samples was collected mid-stream. The donor sample was centrifuged to collect the exfoliated cells. The collected cells were cultured in a medium consisting of DMEM/F12 medium (Gibco) supplemented with 10% FBS (Gibco), 0.1 mM non-essential amino acids (NEAA), 1 mM GlutaMAX (Life Technologies), 0.1 mM β-mercaptoethanol, and SingleQuot Kit CC-4127 REGM (Lonza).

When the urine cells were amplified to a sufficient quantity, an episomal pCEP4 vector that contained the miR302–367 precursor and the other pCEP4 vector that carried OCT4, KLF4, SOX2, and SV40LT genes were simultaneously transfected into the urine cells via nucleofection (Amaxa Basic Nucleofector Kit for primary mammalian epithelial cells, T-013 program, Lonza). The transfected urine cells were cultured in Matrigel-coated 6-well plates (1 × 10^5^–3 × 10^5^ cells per well) with the urine cell culture medium for the first 2 days. The medium was changed to mTeSR1 and refreshed every 2 days for the remaining 13 days. Cell colonies were picked up and transferred to a new Matrigel-coated plate with mTeSR1 and Y-27632 (10 µM, Selleckchem). The culture medium was changed daily to fresh mTeSR1. The cells were dissociated to single cells for further cell expansion. Expression of pluripotent markers Sox2, Nanog and Oct4 was confirmed by immunofluorescence. Karyotype analysis was performed to identify whether the FPD patient iPSCs had normal karyotype. In vivo pluripotency was evaluated with teratoma analyses. The iPSCs derived from the FPD patient with PLA2G6^D331Y/D331Y^ mutation were referred to as PLA2G6^D331Y/D331Y^ iPSCs.

### DA neuron differentiation

FP-cell-based DA neuron induction was performed as previously described, with some minor modifications^46,47^. iPSCs were disaggregated using Accutase for 2 min, centrifuged at 200 × *g* for 3 min, plated on Matrigel-coated multiwells in the presence of Y-27632 at 2 × 10^5^ cells/cm^2^, and then incubated with FP cell induction medium N1 containing SB431542 (10 μM; Tocris) and LDN193189 (100 nM; Miltenyi Biotec), SHH-C24 (100 ng/mL; Peprotech), FGF8 (100 ng/mL; Peprotech), and purmorphamine (2 μM; Tocris) from day 0 to 5. At day 3 to 11, CHIR99021 (3 μM; Tocris) was added to the culture. N1 medium was gradually shifted to N2 medium starting on day 5 of differentiation, by mixing N1 and N2 in ratios of 75% (N1): 25% (N2) on days 5 and 6, 50% (N1): 50% (N2) on days 7 and 8, and 25% (N1): 75% (N2) on days 9 and 10. On day 11, cells were passaged for expansion and cryopreservation. N1 medium (50 mL) contains 41 mL Knockout DMEM, 7.5 mL Knockout serum replacement, 0.5 mL GlutaMAX, 0.5 mL NEAAs, and 0.5 mL penicillin/streptomycin. N2 medium (50 mL) contained 48.5 mL DMEM/F12 with HEPES buffer/Neural basal, 0.5 mL N2 supplement, 0.5 mL GlutaMAX, and 0.5 mL penicillin/streptomycin. DA neuron differentiation medium (50 ml) contained 48 mL Neurobasal medium, 1 mL B27, 0.5 mL GlutaMAX, and 0.5 mL penicillin/streptomycin.

After passage 6 expansion, FP cells were disaggregated with Accumax to fresh Matrigel-coated plate, and medium was changed to NB/B27 supplemented with GDNF (PeproTech, 20 ng/mL), BDNF (PeproTech, 20 ng/mL), 0.2 mM ascorbic acid (Sigma–Aldrich), DAPT (10 nM; Tocris), cAMP (10 μM, Sigma–Aldrich), and transforming growth factor β3 (1 ng/mL; R&D). On day 8, cells were dissociated using Accumax and replated on dishes pre-coated with Poly-D-lysine hydrobromide (PDL) and laminin in differentiation medium (NB/B27 + BDNF, AA, GDNF, dbcAMP, TGFP3, and DAPT until the desired maturation stage for a given experiment.

### Immunostaining

The cells were fixed in 4% paraformaldehyde at room temperature for 20 min, blocked with 5% donkey serum in 0.3% Triton X-100 for 1 h, and then incubated with primary antibodies overnight at 4 °C. After washing three times with DPBS, the cells were incubated at room temperature for 1 h with fluorophore-conjugated secondary antibodies (Life Technologies) against the immunoglobulin of the species from which the primary antibody was generated. Upon completion of immunostaining, the cells were stained with DAPI to reveal the cell nuclei. After washing with DPBS, fluorescence was visualized and photographed using Leica confocal microscopy.

### CCK-8 assay

Cell viability was determined by using the CCK-8 assay. Neuronal cells were seeded into 96-well plates (5 × 10^3^ cells/well). After drug treatment, cells were incubated with 10% CCK-8 (dissolved in culture medium) for 1 h. Absorbance at 450 nm was measured using SpectraMax M5 microplate reader. All values were normalized to the control group. The measurement was performed by an investigator who was blind to the experiment.

### Western blotting

Cells in culture plates were rinsed once with ice-cold DPBS and lysed in RIPA buffer containing 1% phenylmethylsulfonyl fluoride and 1% protease/phosphatase inhibitor cocktail (Thermo Fisher Scientific) for 30 min at 4 °C, followed by centrifugation at 12,500 × *g* for 20 min at 4 °C. Lysates in 1× sample buffer were boiled for 5 min at 95 °C for denaturation and separated by SDS-PAGE. The target proteins were detected by western blotting with their respective specific antibodies, and α-tubulin was used as an internal control. The blot was visualized using an ECL kit (GE Healthcare) according to the manufacturer’s instructions. The intensity of the bands was quantified using Image Lab 5.0 software. The quantification was performed by an investigator who was blind to the experiment.

### Measurement of intracellular ROS levels

Intracellular ROS was determined using ROS probe CellROX® Green Reagent (Thermo Fisher Scientific). The harvested cells were incubated with CellROX® Green Reagent (5 µM) for 30 min in the dark. The cells were rinsed twice with DPBS, and then analyzed with BD Accuri C6 cytometry. All values were normalized to the control group. The measurement was performed by an investigator who was blind to the experiment.

### Measurement of mitochondrial membrane potential

The mitochondrial membrane potential was measured by JC-1 staining (Thermo Fisher Scientific). The harvested cells were incubated with JC-1 dye (10 μg/mL in medium) at 37 °C for 30 min. After that, cells were rinsed twice with DPBS, and then analyzed with BD Accuri C6 cytometry. Mitochondrial membrane potential was calculated as the ratio of JC-1 red/green fluorescence intensity. The fluorescent signal in DA neurons was also imaged with a fluorescent microscope. All values were normalized to the control group. The measurement was performed by an investigator who was blind to the experiment.

### Quantification of mitochondrial size

Mitochondrial morphology was determined by MitoTracker Red staining. Neurons were stained with 500 nM MitoTracker Red (Molecular Probes) in culture medium for 30 min at 37 °C, and washed with DPBS. Mitochondrial morphology was imaged by fluorescent microscopy. The mitochondrial size was assessed using Image-J software. All values were normalized to the control group. The quantification was performed by an investigator who was blind to the experiment.

### Calcium imaging

Intracellular Ca^2+^ levels were measured using probe Fluo-4-AM (Beyotime). Cells were incubated with 5 μM Fluo-4-AM at 37 °C in 5% CO_2_. After 1 h, carefully rinsed the cells with Ca^2+^-free extracellular working solution (130 mM NaCl, 4.6 mM KCl, 2 mM MgCl_2_, 10 mM HEPES/Na, 5 mM glucose, 100 mM EGTA, pH 7.4). Time-lapse fluorescence images were recorded by Leica LAX confocal microscopy and analyzed with Leica LAX software. The settings were all the same (FITC/ offset/ Pinhole/ zoom in or zoom out). During data capture, the baseline cytosolic Ca^2+^ concentration was recorded for 8 min, and at 9 min, TG (0.3 μM) was added to block SERCA and induce acute calcium leakage from the ER. Additional Ca^2+^ (2 mM) was added at 22 min to evoke recruitment of ER and cytosolic Ca^2+^ via SOCE. All values were normalized to the control group. The investigator who performed calcium image acquisition and image analysis was blind to the experiment.

### Statistical analysis

Statistical analysis was performed using GraphPad Prism 5.0 statistical software. All experiments were independently performed in triplicate. Data were expressed as mean ± SEM. Statistical analysis was carried out using one-way analysis of variance followed by Tukey’s multiple comparison or two-sided Mann–Whitney U test for two groups. A *p*-value <0.05 was considered significant.

## Supplementary information

Suppl. Fig 1

Suppl. Fig 2

Suppl. Fig 3

Supplementary figure legends
