# Supplementary figures and images for: Azoramide protects iPSC-derived dopaminergic neurons with PLA2G6 D331Y mutation through restoring ER function and CREB signaling
Source: Cell Death Dis. 2020 Feb 18;11(2):130. doi: 10.1038/s41419-020-2312-8 (PMC7028918; doi:10.1038/s41419-020-2312-8)

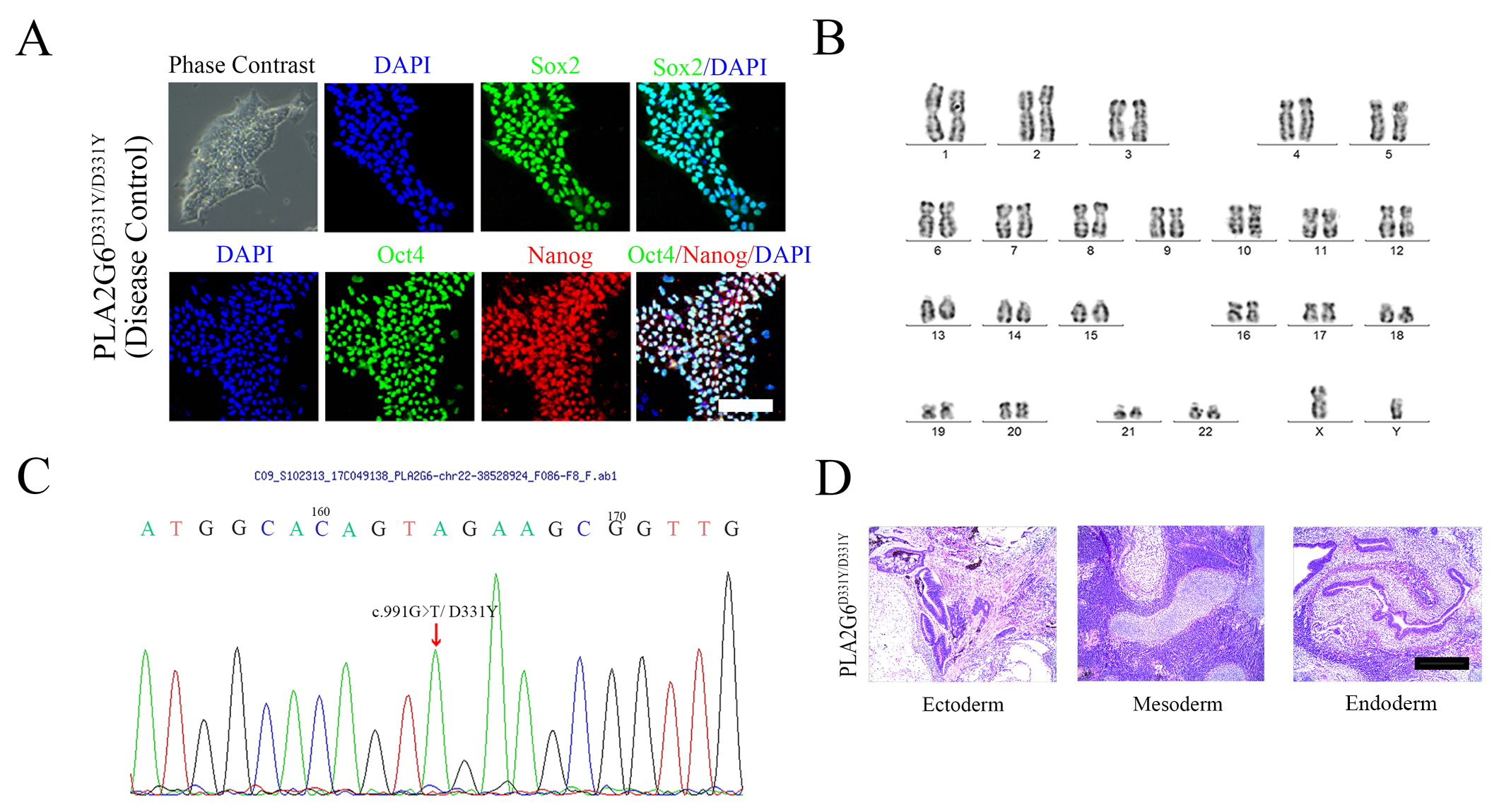

Supplement: Supplementary file 1 — Suppl. Fig 1 [file 41419_2020_2312_MOESM1_ESM.tif]

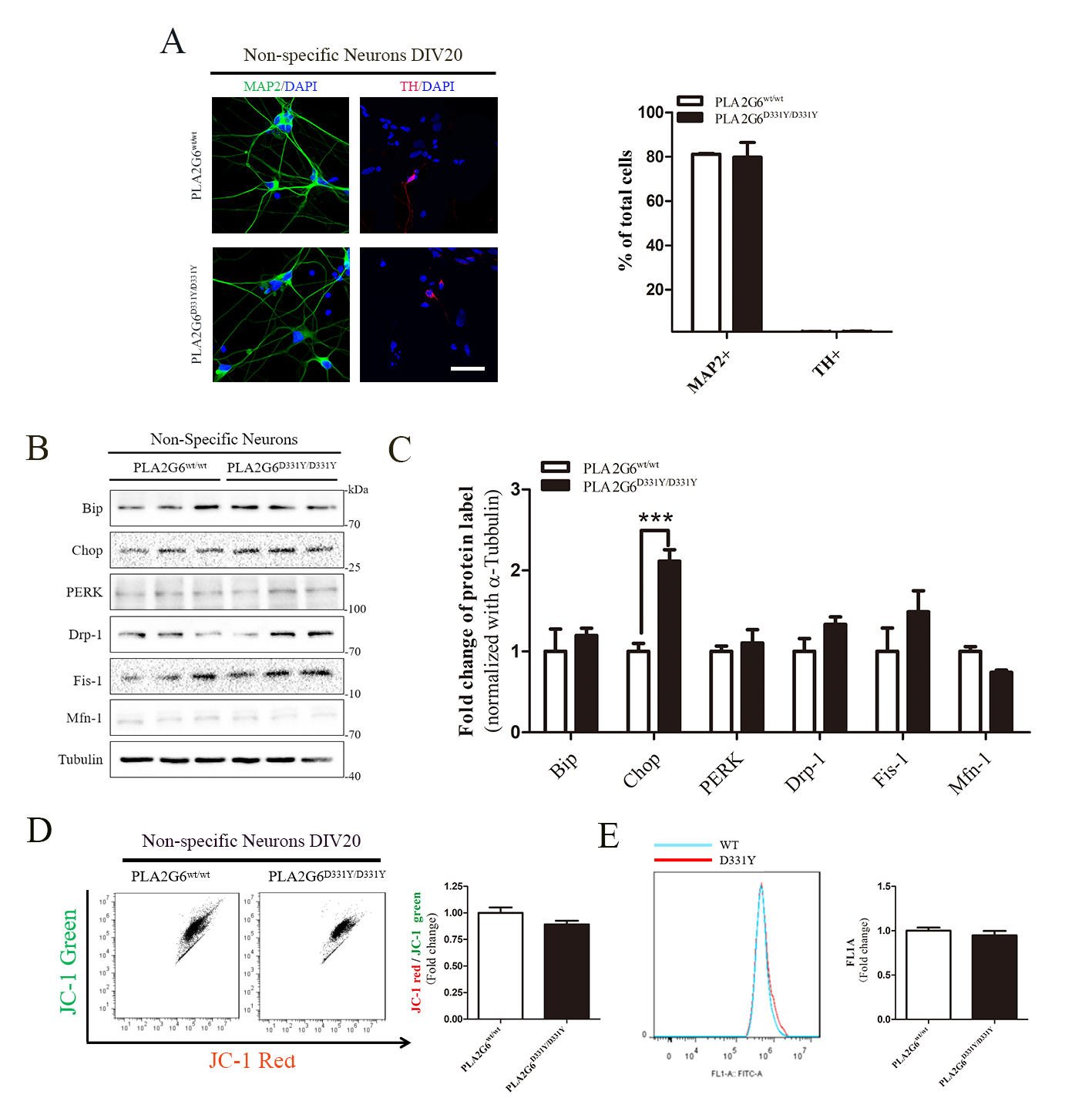

Supplement: Supplementary file 2 — Suppl. Fig 2 [file 41419_2020_2312_MOESM2_ESM.tif]

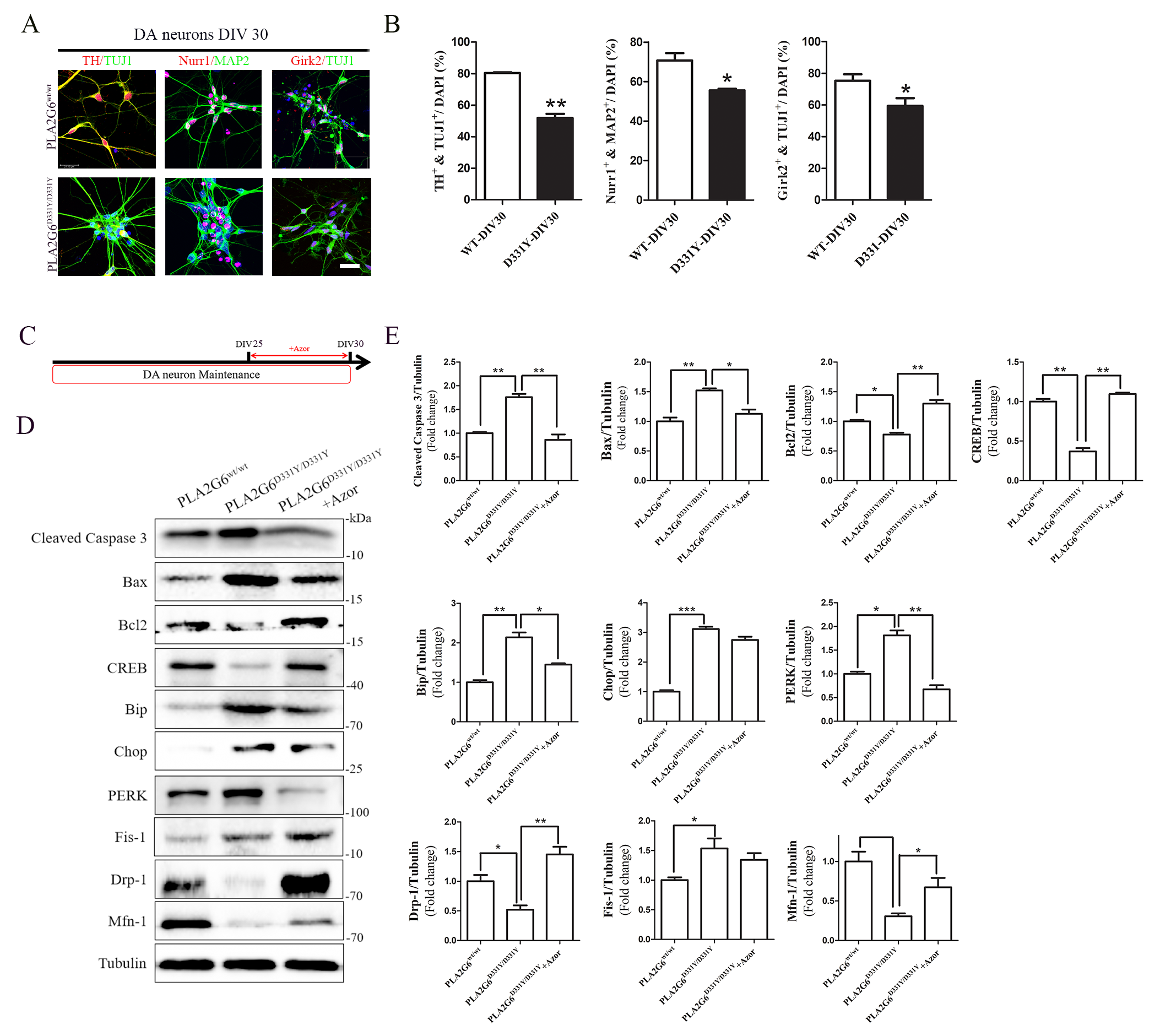

Supplement: Supplementary file 3 — Suppl. Fig 3 [file 41419_2020_2312_MOESM3_ESM.tif]
