# Supplementary material for: Azoramide protects iPSC-derived dopaminergic neurons with PLA2G6 D331Y mutation through restoring ER function and CREB signaling
Source: Cell Death Dis. 2020 Feb 18;11(2):130. doi: 10.1038/s41419-020-2312-8 (PMC7028918; doi:10.1038/s41419-020-2312-8)
Supplement: Supplementary file 4 — Supplementary figure legends [file 41419_2020_2312_MOESM4_ESM.docx]

**Azoramide protects iPSC-derived dopaminergic neurons with PLA2G6 D331Y mutation through restoring ER function and CREB signaling**

Minjing Ke^1, #^, Cheong-Meng Chong^1, #^, Haitao Zeng^2^, Miaodan Huang^1^, Zhijian Huang^1^, Ke Zhang^1^, Xiaotong Cen^3^, Jia-Hong Lu^1^, Xiaoli Yao^4^, Dajiang Qin^3*^, Huanxing Su^1*^

^1^State Key Laboratory of Quality Research in Chinese Medicine, Institute of Chinese Medical Sciences, University of Macau, Macao, China

^2^Center for Reproductive Medicine, the Sixth Affiliated Hospital of Sun Yat-Sen University, Guangzhou 510080, China

^3^South China Institute for Stem Cell Biology and Regenerative Medicine, Guangzhou Institute of Biomedicine and Health, Chinese Academy of Sciences, Guangzhou, China

^4^Department of Neurology, National Key Clinical Department and Key Discipline of Neurology, the First Affiliated Hospital of Sun Yat-Sen University, Guangzhou 510080, China

^#^These authors contributed equally to this work.

***Correspondence to:**

Huanxing Su, State Key Laboratory of Quality Research in Chinese Medicine, Institute of Chinese Medical Sciences, University of Macau, Macao, China.

Tel: (853) 8822 8518; Fax: (853) 2884 1358

Email: [huanxingsu@um.edu.mo](mailto:huanxingsu@um.edu.mo)

Dajiang Qin, South China Institute for Stem Cell Biology and Regenerative Medicine, Guangzhou Institutes of Biomedicine and Health, Chinese Academy of Sciences, Guangzhou, China

Email: [qin_dajiang@gibh.ac.cn](mailto:qin_dajiang@gibh.ac.cn)

**Supplementary Figure Legends:**

Suppl. Fig 1. Characterization of FPD PLA2G6 D331Y mutant iPSC. (A) Immunostaining of PLA2G6wt/wt and PLA2G6D331Y/D331Y iPSCs for Nanog, Sox2, and Oct4. (B) Karyotype analysis of PLA2G6 D331Y iPSCs. (C) Genotype analysis showed that there was one homozygous mutation in exon 7 of PLA2G6 gene: C.991G >t (the coding region no.991 nucleotide was mutated from G to T), leading to amino acid change p.d331y (amino acid no. 331 was mutated from aspartic acid to tyrosine), which was a missense mutation. (D) Terotoma analysis of PLA2G6 D331Y iPSCs. Scale bar: 100 µm for A and 250 µm for D.

Suppl. Fig 2. No obvious ER stress and mitochondrial dysfunction were observed in non-dopaminergic neurons with PLA2G6 mutant (A) Immunostaing showing non-dopaminergic neurons were MAP2-positive but TH-negative. (B-C): Western blotting demonstrates that no elevated expresion levels of UPR and mitochondria related proteins in non-dopaminergic neurons were found between the PLA2G6 mutant group and the control group except that the expresion level of Chop was significanlty increased in PLA2G6 mutant neurons. (D) Mitochondrial membrane potential was determined by JC-1 staining. (E) Intracellular ROS levels were measured by the probe CellROX® Green Reagent and flow cytometry. Data are represented as mean ± SEM. All experiments were replicated in triplicate independently. **p < 0.01, ***p < 0.005. Scale bar: 20 µm.

Suppl. Fig 3. Azoramide protects PLA2G6 mutant dopaminergic neurons after culture of 30 days. (A-B) Immunostaining shows that the percentage of the cells expressing TH, Nurr1, and Girk was significantly decreased in PLA2G6 mutant group compared to the control group. (C) Schematic diagram of drug treatment. (D-E) Western blotting demonstrated that azoramide significantly decreased the cleaved level of caspase 3 and the ratio of Bax/Bcl2 and enhanced the expression of CREB in PLA2G6 mutant neurons. Meanwhile, azoramide significantly suppressed the expression of UPR proteins, elevated the expression level of mfn1, and inhibited the expression levels of DRP1 and Fis1 in PLA2G6 mutant neurons. Data are represented as mean ± SEM. All experiments were replicated in triplicate independently. **p < 0.01, ***p < 0.005. Scale bar: 20 µm.
